# Papaverine Targets STAT Signaling: A Dual‐Action Therapy Option Against SARS‐CoV‐2

**DOI:** 10.1002/jmv.70319

**Published:** 2025-04-02

**Authors:** Philipp Reus, Emma Torbica, Tamara Rothenburger, Marco Bechtel, Joshua Kandler, Sandra Ciesek, Philip Gribbon, Aimo Kannt, Jindrich Cinatl, Denisa Bojkova

**Affiliations:** ^1^ Fraunhofer Institute for Translational Medicine and Pharmacology ITMP, ScreeningPort Hamburg Germany; ^2^ Institute for Medical Virology, University Hospital, Goethe University Frankfurt Frankfurt Germany; ^3^ Fraunhofer Institute for Translational Medicine and Pharmacology ITMP Frankfurt Germany; ^4^ Dr. Petra Joh Research Institute Frankfurt Germany

**Keywords:** antiviral, immunomodulatory, interferon, papaverine, SARS‐CoV‐2

## Abstract

Papaverine (PV) has been previously identified as a promising candidate in SARS‐CoV‐2 repurposing screens. In this study, we further investigated both its antiviral and immunomodulatory properties. PV displayed antiviral efficacy against SARS‐CoV‐2 and influenza A viruses H1N1 and H5N1 in single infection as well as in co‐infection. We demonstrated PV's activity against various SARS‐CoV‐2 variants and identified its action at the post‐entry stage of the viral life cycle. Notably, treatment of air‐liquid interface (ALI) cultures of primary bronchial epithelial cells with PV significantly inhibited SARS‐CoV‐2 levels. Additionally, PV was found to attenuate interferon (IFN) signaling independently of viral infection. Mechanistically, PV decreased the activation of the IFN‐stimulated response element following stimulation with all three IFN types by suppressing STAT1 and STAT2 phosphorylation and nuclear translocation. Furthermore, the combination of PV with approved COVID‐19 therapeutics molnupiravir and remdesivir demonstrated synergistic effects. Given its immunomodulatory effects and clinical availability, PV shows promising potential as a component for combination therapy against COVID‐19.

## 1 Introduction

The etiologic agent of COVID‐19, SARS‐CoV‐2, has caused the long‐lasting and still ongoing pandemic that significantly impacted the recent years. While various vaccines are available, they offer only limited protection against newly emerging virus variants and hospitalization rates remain substantial [1]. A contributing factor to severe COVID‐19 disease is the dysregulated innate immune response that can culminate in cytokine storm‐induced acute respiratory distress syndrome [2]. Most of the currently approved small molecule drugs exhibit limited efficacy, the potential for resistance development or the risk of disease rebound [3, 4], highlighting the ongoing need for effective antiviral treatment against COVID‐19. Combination therapy can be a promising approach to improve drug efficacy as well as optimize pharmacokinetic/pharmacodynamic (PK/PD) properties, as evidenced by clinical trials with Paxlovid or Evusheld (Clinical trials NCT04960202 and NCT04625725).

Papaverine (PV), an alkaloid from opium poppy, is an approved drug for treatment of different (cardio)‐vascular conditions [5, 6] and has been found to relieve vasospasm by dilating and relaxing the smooth muscles. Furthermore, its anticancer activity [7, 8], anti‐inflammatory properties [9, 10, 11] and activity against different types of viruses like ortho‐ and paramyxoviruses [12] have been previously described. Mechanistically, PV targets phosphodiesterase (PDE) activity, mainly PDE10A, but also PDEs 3A, 4 and 6 [13, 14]. PV has been identified as active against SARS‐CoV‐2 in a repurposing screening [15], and its potential as a COVID‐19 drug has previously been suggested [16], however, its inhibitory effect in the context of SARS‐CoV‐2 has not yet been thoroughly characterized.

In this study, we examined the anti‐SARS‐CoV‐2 activity of PV and its derivatives, ethaverine (EV) and drotaverine (DV), using various cell models. Notably, we discovered novel immunomodulatory properties of PV, which suppresses the activation and translocation of STAT1 and STAT2. This dual effect of PV, both antiviral and immunosuppressive, along with its potential in combination therapy, has not been previously demonstrated and highlights the potential of this established drug for COVID‐19 treatment.

## 2 Materials and Methods

### 2.1 Cell Culture

Caco‐2‐F03 (Resistant Cancer Cell Line collection [17]) and Calu‐3 (ATCC) cells were cultured in Minimal Essential Medium or Iscove's Modified Dulbecco's Medium, respectively, both supplemented with 10% fetal bovine serum (FBS), 100 IU/mL penicillin, 100 μg/mL streptomycin, and 2 mM glutamine. HEK‐Blue ISG, A549‐Dual hACE2‐TMPRSS2, A549‐Dual KO‐RIG‐I hACE2‐TMPRSS2, and A549‐Dual KO‐MDA5‐I hACE2‐TMPRSS2 cells (Invivogen) were cultured in Dulbecco's Modified Eagle's Medium supplemented with 10% FBS, 100 IU/mL penicillin, 100 μg/mL streptomycin, and 2 mM glutamine as well as 100 µg/mL zeocin (for HEK‐Blue cells) or 10 µg/mL blasticidin, 100 µg/mL hygromycin, 0.5 µg/mL puromycin, and 100 µg/mL zeocin (for all three A549 Dual cell lines) for cell selection. Infection experiments were carried out in the corresponding media containing only 1% FBS.

Primary human bronchial epithelium cells (HBEpCs) were isolated from the lung explant tissue of a patient with lung emphysema, as previously described [18]. For differentiation into air‐liquid interface (ALI) cultures, the cells were resuscitated, passaged once in PneumaCult‐Ex Medium (STEMCELL Technologies) and subsequently seeded on transwell inserts (Sarstedt). After becoming fully confluent, the apical medium was removed, and the basal medium was replaced with PneumaCult ALI Maintenance Medium (STEMCELL Technologies), supplemented with Antibiotic/Antimycotic solution (Sigma Aldrich) and MycoZap Plus PR (Lonza). The medium was changed, and cells were washed with phosphate‐buffered saline every other day. Successful differentiation was deemed as an increasing transepithelial electrical resistance, development of ciliation, and the production of mucus.

### 2.2 Virus Strains

All used SARS‐CoV‐2 strains were isolated from patient material, amplified in Caco‐2‐F03 cells and stored at −80°C until use. The following SARS‐CoV‐2 strains have been used in this study: D614G (SARS‐CoV‐2/FFM7, GenBank: MT358643), alpha (SARS‐CoV‐2/FFM‐UK7931/2021, GenBank: MZ427280), beta (SARS‐CoV‐2/FFM‐ZAF1/2021, GenBank: MW822592), delta (SARS‐CoV‐2/FFM‐IND8424/2021, GenBank: MZ315141), omicron BA.1 (SARS‐CoV‐2/FFM‐SIM0550/2021, GenBank: OL800702).

The influenza A virus (IAV) strains A/Puerto Rico/8/34(H1N1) and A/Hong Kong/213/03(H5N1) were supplied by ATCC (VR‐1469) and the World Health Organization (WHO) Influenza Center (National Institute for Medical Research, London, UK), respectively, amplified in MDCK cells (ATCC, CCL‐34) in medium supplemented with 2 µg/mL of trypsin and subsequently stored at −80°C until use. All viral titers were determined via TCID_50_ assay.

### 2.3 Antiviral Assays

For immunofluorescence imaging, confluent layers of Calu‐3 were pretreated with PV for 1 h and subsequently infected with SARS‐CoV‐2 (delta variant, MOI 0.01, 48 h), H1N1 (MOI 0.01, 24 h), H5N1 (MOI 0.001, 24 h) or both SARS‐CoV‐2 and H1N1 (both MOI 0.1). After fixation with methanol/acetone (60:40), the cells were blocked with 5% goat serum and 2% BSA and stained using a primary anti‐SARS‐CoV‐2 spike S1 (1:1500, Sino Biological, #40150‐R007) and/or anti‐IAV nucleoprotein (1:1500, Sigma Aldrich, #MAB8251) antibody, respectively. The secondary staining, Alexa Fluor 647 (1:1000, ThermoFisher Scientific, #A‐21246) and Alexa Fluor 488 (1:1000, ThermoFisher Scientific, #A‐11001) antibodies, as well as DAPI (0.2 µg/mL, Carl Roth, #6335.1), were used, and cells were imaged and analyzed via Operetta CLS High Content Analysis System and Harmony software (Revvity).

For immunocytochemistry analyses, confluent layers of Calu‐3 or Caco‐2 cells were pretreated with PV, EV, or DV for 1 h and subsequently infected with different SARS‐CoV‐2 variants (D614G, alpha, beta, delta, or omicron BA.1 variant, MOI 0.01, 24 and 48 h for Caco‐2 and Calu‐3, respectively). After fixation with methanol/acetone (60:40), the cells were blocked with 5% goat serum and 2% BSA and stained using a primary anti‐SARS‐CoV‐2 spike S1 (1:1500, Sino Biological, #40150‐R007) and secondary anti‐rabbit peroxidase‐coupled antibody (1:1000, Jackson ImmunoResearch, #111‐035‐144). Acquisition and quantification of the signal were done via incubation with AEC substrate and measurement via Bioreader 7000‐F‐Z‐I microplate reader (Biosys). The results are expressed as the percentage inhibition compared to an untreated, infected control.

### 2.4 Cytotoxicity Assays

To determine cytotoxic concentrations of PV, EV, and DV, confluent layers of Caco‐2 or Calu‐3 cells were treated with the drugs for 24 or 48 h, respectively, fixed with methanol/acetone (60:40), stained with 0.04% sulforhodamine B (SRB) solution (protocol adapted from Orellana and Kasinski [19]), and the absorbance was measured at 510 nm. The results are expressed as the percentage viability compared to an untreated control.

### 2.5 Time of Addition Assays

For the time of addition experiments, Calu‐3 cells were infected with SARS‐CoV‐2 (delta variant, MOI 2) and treated with PV, EV, or DV according to two different schemes: The first group was treated only during the initial hour of virus infection, after which the virus and the compounds were removed and incubated in medium for the remainder of the experiment. The second group was treated after the initial hour of virus infection, after which the virus was removed, and the compounds were added for the remainder of the experiment. After 8 h, the cells from both treatment groups were lysed, and intracellular RNA was extracted using the RNeasy Mini Kit (Qiagen). The virus RNA amounts were quantified via qRT‐PCR with primers against the SARS‐CoV‐2 E gene (fwd: ACAGGTACGTTAATAGTTAATAGCGT; rev: ATATTGCAGCAGTACGCACACA; adapted from a WHO protocol [20]) using the Luna Universal One‐Step RT‐qPCR Kit (New England Biolabs) and a CFX96 Real‐Time System, C1000 Touch Thermal Cycler (Bio‐Rad). GAPDH (fwd: AAGGCTGGGGCTCATTTGCAG; rev: GCAGGAGGCATTGCTGATGATC) was used as a reference gene, and the values for E gene expression were normalized to the virus control, which was infected but received no treatment.

### 2.6 Infection Assay in HBEpCs

To assess the antiviral activity of PV in HBEpCs growing on transwells under ALI conditions, the cells were infected with SARS‐CoV‐2 (omicron BA.1 variant, MOI 1) for 2 h on the apical side before removal of the virus and addition of compound dilutions on both the apical and basal side. After a further 10 h, the apical medium was removed. 72 h post infection, RNA from apical washes was isolated via QIAamp Viral RNA Kit (Qiagen) and virus copy number was determined by Luna Universal One‐Step RT‐qPCR Kit (New England Biolabs), using RdRp primers (fwd: GTGARATGGTCATGTGTGGCGG; rev: CARATGTTAAASACACTATTAGCATA; adapted from a WHO protocol [20]) and an RdRp standard curve. Basal medium was collected for cytotoxicity assessment via LDH release assay. Lastly, the cells were collected and lysed for immunoblot analysis (see Section 2.7).

### 2.7 Immunoblot Analysis

Whole‐cell lysates of Calu‐3 cells were prepared using a Triton‐X lysis buffer containing “cOmplete protease inhibitor cocktail” (Roche). Protein concentrations were assessed via the “DC Protein assay” (Bio‐Rad Laboratories). Proteins were separated by sodium dodecyl sulfate‐polyacrylamide gel electrophoresis and transferred to nitrocellulose membranes (ThermoFisher Scientific). For protein detection the following primary antibodies were used: GAPDH (1:1000, R&D Systems, #2275‐PC‐020), ISG15 (1:200, Santa Cruz, #sc166755), MX1 (1:1000, Cell Signaling, #37849), pSTAT1 Y701 (1:1000, Cell Signaling, #9167), pSTAT2 Y689 (1:1000, Upstate, #02‐224), SARS‐CoV‐2 Nucleoprotein (1:1000, Sino Biological, #40143‐R019), STAT1 (1:1000, Cell Signaling, #14994), STAT2 (1:1000, Upstate, #06‐502) and Tubulin (1:1.000, Abcam, #ab179513). Protein bands were visualized using IRDye‐labeled secondary antibodies at dilution 1:40 000 (LI‐COR Biotechnology, IRDye 800CW Goat anti‐Rabbit, #926‐32211 and IRDye 800CW Goat anti‐Mouse IgG, #926‐32210) and Odyssey Infrared Imaging System (LI‐COR Biosciences).

### 2.8 Interferon (IFN) Release Assay

To measure the release of type I, II and III IFNs, Calu‐3 cells were treated with PV and 1 µg/mL poly I:C for 24 h and their supernatants subsequently incubated with HEK‐Blue reporter cells for IFN α/β (type I), γ (type II), and λ (type III) for another 24 h. IFN release, as an expression of reporter activity, was measured via QuantiBlue Solution (Invivogen) at 640 nm. The results are expressed as the percentage of IFN release, normalized to the respective stimulated controls which received no drug.

### 2.9 IFN‐Stimulated Response Element (ISRE) Activation Assay

To investigate the effect of PV treatment on ISRE activation, HEK‐Blue ISG cells (Invivogen), containing a SEAP gene coupled to ISRE, were treated with PV or baricitinib (BAR) and subsequently stimulated with either type I, II, or III IFN (IFN β, IFN γ, and IFN λ3, respectively) for 24 h. Supernatants of the cells were then incubated with QuantiBlue Solution, and the signal was detected at 640 nm. The results are expressed as a percent of ISRE activation normalized to the respective stimulated controls which received no drug.

Similarly, A549‐Dual hACE2‐TMPRSS2, A549‐Dual KO‐RIG‐I hACE2‐TMPRSS2 and A549‐Dual KO‐MDA5‐I hACE2‐TMPRSS2 cells (Invivogen), carrying both an ISRE and nuclear factor “kappa‐light‐chain‐enhancer” of activated B‐cells (NFκB) reporter, were treated with PV and 1 µg/mL poly I:C for 24 h and subsequently incubated with QuantiBlue or QuantiLuc Solution (Invivogen) and absorbance at 640 nm or luminescence were measured, respectively. The results are expressed as a percent of ISRE or NFκB reporter activity and are normalized to the respective stimulated controls which received no drug.

### 2.10 STAT Translocation Assay

HEK‐Blue ISG cells were either mock‐treated or treated with PV or BAR, stimulated with 1 × 10^3^ U/mL of IFN β for 15, 30 min, 1, or 4 h and fixed with methanol/acetone (60:40). Phosphorylation and translocation of STAT1 and STAT2 were investigated via immunofluorescence staining using the antibodies for STAT1, pSTAT1, STAT2, and pSTAT2 that have also been used in the immunoblot analyses as well as DAPI (0.2 µg/mL, Carl Roth, #6335.1) for visualization of nuclei. For the analysis, the cell populations with nuclear pSTAT1/2 signal were determined using an intensity threshold, set based on the mock control and untreated, but IFN‐stimulated control. In addition, the fluorescence intensities of the nucleus and cytoplasm areas of the cells were determined.

### 2.11 Drug Combination Assays

For the assessment of drug synergies, Calu‐3 cells were pretreated with PV in combination with either nirmatrelvir (NIR) (the main active ingredient of Paxlovid), EIDD‐1931 (EIDD) (the active metabolite of the pro‐drug molnupiravir) or remdesivir (REM) at different concentrations, in the presence of a P‐glycoprotein inhibitor (P‐gp_i_) to prevent drug efflux, 1 h before infection with SARS‐CoV‐2 (delta variant, MOI 0.01). After 48 h, the cells were fixed with methanol/acetone (60:40), were blocked with 5% goat serum and 2% BSA and stained using a primary anti‐SARS‐CoV‐2 spike S1 (1:1500, Sino Biological, #40150‐R007) and secondary anti‐rabbit peroxidase‐coupled antibody (1:1000, Jackson ImmunoResearch, #111‐035‐144). Acquisition and quantification of the signal were done via incubation with AEC substrate and measurement via Bioreader 7000‐F‐Z‐I microplate reader (Biosys). The combinations were then assessed for potential synergistic effects using the SynergyFinder software (as described in Ianevski, Giri and Aittokallio [21]) based on a Zero interaction potency (ZIP) reference model. Deviations between observed and expected responses denote synergism, for positive values > 10, or antagonism, for negative values < −10 [22].

### 2.12 Statistics

All results are expressed as the mean ± standard deviation (SD) from three independent experiments unless stated otherwise. Statistical significance is displayed with their *p*‐value directly above the corresponding bars. The statistical tests conducted are mentioned in the respective figure legends. Curve fittings and IC_50_ value calculations were conducted with the formula “Sigmoidal, 4PL, X is concentration” of GraphPad Prism 9.

## 3 Results

### 3.1 PV and Its Derivatives Exhibit Antiviral Activity Against Both SARS‐CoV‐2 and IAV H1N1

**Figure 1:**
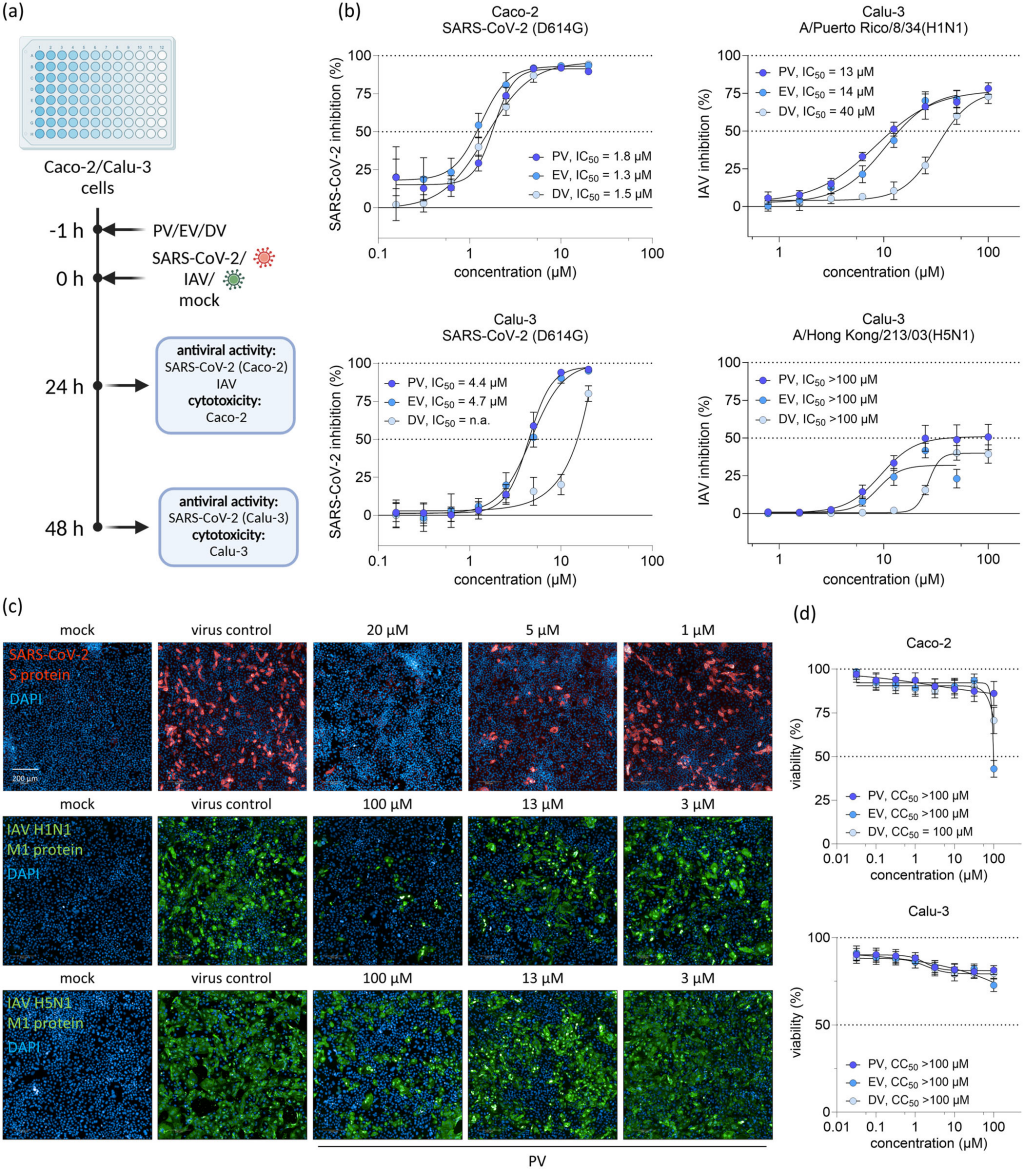
PV and its derivatives exhibit antiviral activity against both SARS‐CoV‐2 and IAV H1N1. (a) Experimental scheme of the antiviral and toxicity experiments. Caco‐2 or Calu‐3 cells were pretreated with PV, EV, or DV 1 h before infection with SARS‐CoV‐2 (MOI 0.01) or IAV (H1N1, MOI 0.01 or H5N1, MOI 0.001) for measurement of antiviral activity or mock‐infection for determination of compound toxicity via SRB assay. (b) Dose‐response curves and IC50 values of Caco‐2 and Calu‐3 cells treated with PV, EV, or DV and infected with SARS‐CoV‐2 (D614G variant) or IAV, measured via immunocytochemistry. (c) Representative immunofluorescence images of Calu‐3 cells infected with SARS‐CoV‐2 (Delta variant) or IAV H1N1/H5N1. (d) Cytotoxicity measurement of uninfected Caco‐2 or Calu‐3 cells treated with PV, EV, or DV, measured via SRB assay. Results are expressed as the mean ± SD.

To elucidate potential class‐specific effects, we initially characterized the antiviral efficacy of PV in comparison to its derivatives, DV and EV (Figure 1a). All three compounds showed comparable dose–response effects against the SARS‐CoV‐2 D614G variant, with IC_50_ values of approximately 1.5 µM in Caco‐2 cells (Figure 1b). Notably, PV and EV displayed slightly reduced efficacy in Calu‐3 cells, with IC_50_ values of 4.4 µM and 4.7 µM, respectively, whereas DV did not achieve complete inhibition of SARS‐CoV‐2 even at concentrations as high as 20 µM in this cell line. Subsequently, we assessed the antiviral activity of PV against IAV H1N1, previously shown to be susceptible to PV treatment [12], as well as against IAV H5N1, a virus with significant pandemic potential [23, 24]. The three compounds exhibited reduced activity against IAV H1N1, with IC_50_ values of 13, 14, and 40 µM for PV, EV, and DV, respectively. None of the compounds achieved complete viral inhibition against IAV H5N1 at concentrations up to 100 µM (Figure 1b,c). These IC_50_ values against H1N1 were comparable to the ones described in the study of Aggarwal et al. [12], which reported IC_50_ of 16.77 and 24.54 µM for A/WSN/33 (H1N1) and A/PR/8/34 (H1N1), respectively.

We also conducted an evaluation of cell viability under treatment with PV and its derivatives. All three compounds were largely nontoxic, even at higher concentrations (Figure 1d). However, EV, and to a lesser extent DV, were observed to reduce cell viability at 100 µM in Caco‐2 cells.

### 3.2 PV Is Effective Against SARS‐CoV‐2 and IAV H1N1 Co‐Infection

**Figure 2:**
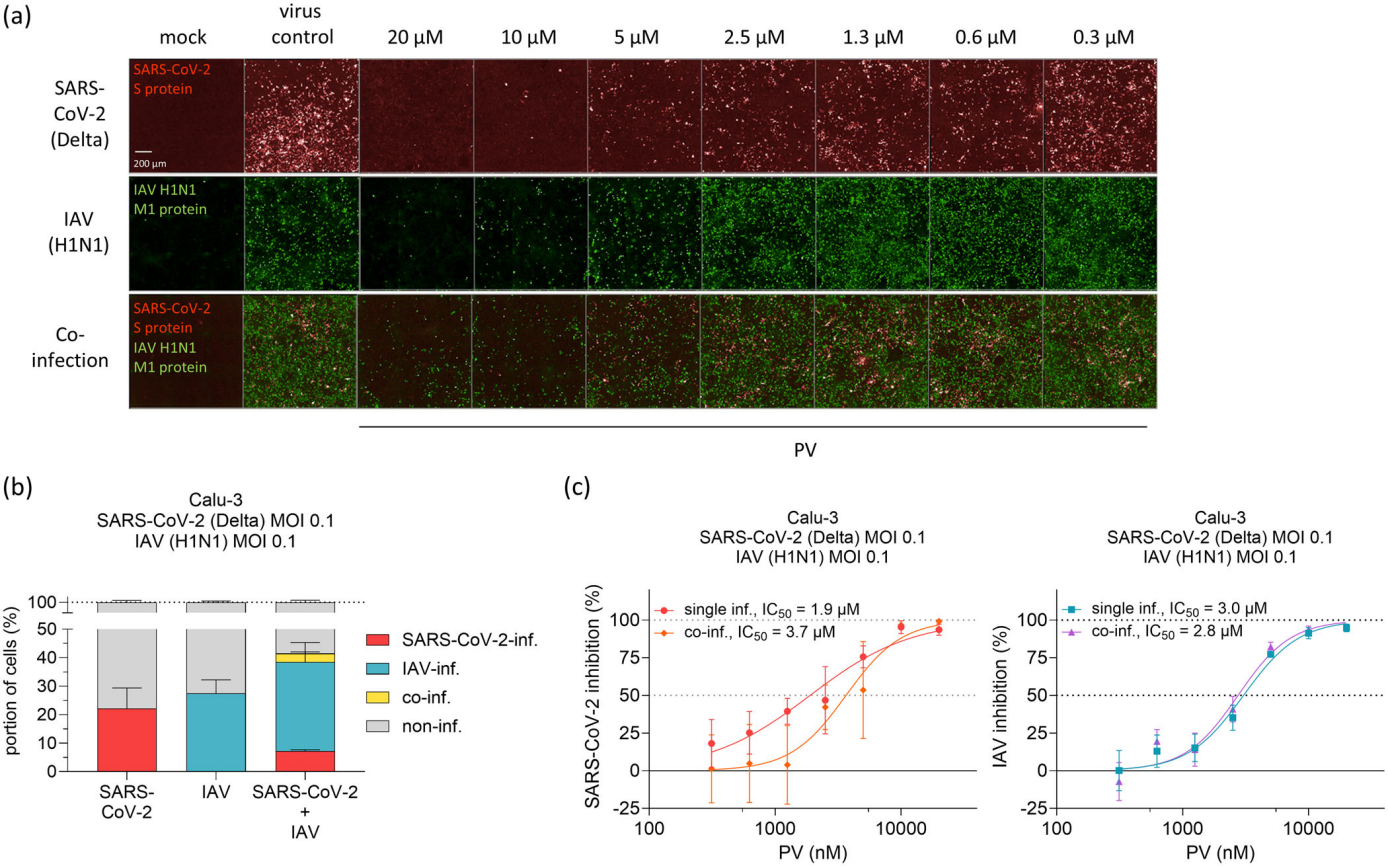
PV is effective against SARS‐CoV‐2 and IAV H1N1 co‐infection. (a) Representative immunofluorescence images of Calu‐3 cells, pretreated with PV 1 h before infection with SARS‐CoV‐2 (Delta, MOI 0.1) and/or IAV (A/Puerto Rico/8/34(H1N1), MOI 0.1) for 24 h for measurement of antiviral activity. (b) SARS‐CoV‐2 and IAV infection levels of untreated Calu‐3 cells after 24 h of infection. (c) Comparison of IC50 values of PV against SARS‐CoV‐2 and IAV in a single versus co‐infection scenario.

Co‐infections with respiratory viruses, such as influenza and SARS‐CoV‐2, are clinically significant as they often lead to more severe illness and complicate patient management [25, 26]. To evaluate PV's antiviral efficacy during co‐infection, we simultaneously infected Calu‐3 cells with the SARS‐CoV‐2 Delta variant and IAV H1N1, followed by PV treatment. SARS‐CoV‐2 showed reduced infection rates in co‐infected, untreated cells (22.2% vs. 7.2% in single‐ vs. co‐infected cells), while IAV infection levels remained mostly unchanged with 27.5% and 31.3%, respectively (Figure 2a,b). Notably, PV effectively inhibited both viruses with minimal to no reduction in efficacy compared to single infections (Figure 2a,c).

### 3.3 PV Inhibits SARS‐CoV‐2 in Airway Epithelium Cells Post‐Entry

**Figure 3:**
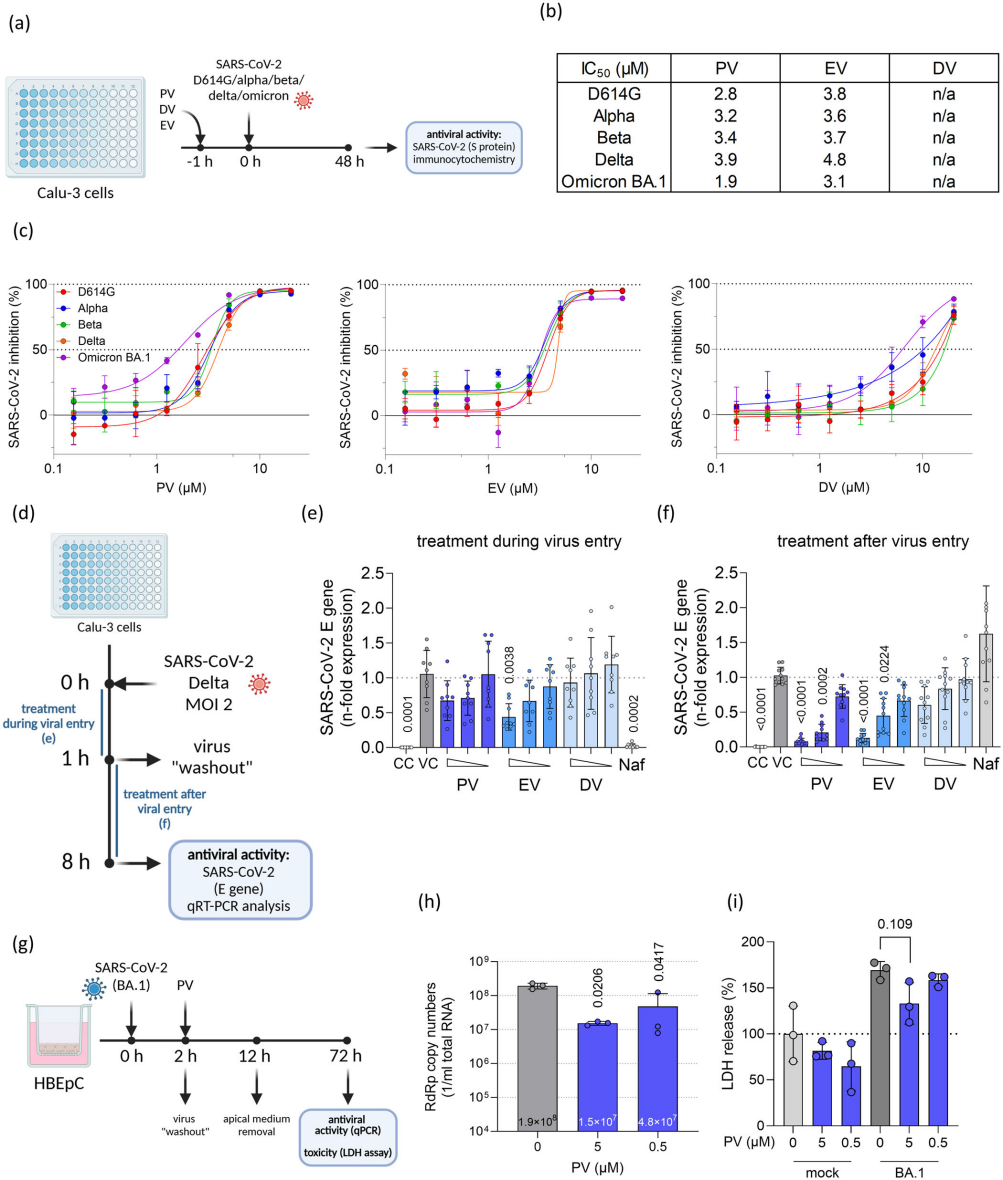
PV inhibits SARS‐CoV‐2 in airway epithelium cells post‐entry. (a) Experimental scheme of the antiviral testing against different virus variants. Calu‐3 cells were pretreated with PV, EV, or DV 1 h before infection with different SARS‐CoV‐2 variants (MOI 0.01). (b) The IC50 values were derived from the dose‐response curves (c) of Calu‐3 cells treated with PV, EV, or DV and infected with different variants of concern, as determined by immunocytochemistry. (d) Experimental scheme of the “time of addition” experiments. Calu‐3 cells were infected with SARS‐CoV‐2 (Delta variant, MOI 2) for 8 h. For condition #1, treatment with PV, EV, or DV occurred only during the first hour of infection (virus entry phase); for condition #2, treatment occurred after entry until end of the experiment 8 h post‐infection. (e) and (f) Levels of SARS‐CoV‐2 E gene RNA after treatment with PV, EV, or DV during (e) or after (f) virus entry, respectively, as determined by qRT‐PCR. (g) Experimental scheme of the antiviral testing of HBEpCs, infected with different SARS‐CoV‐2 (Omicron BA.1 variant, MOI 1) and subsequently treated with PV dilutions. Seventy‐two hours after infection, the cells were processed for further analysis. (h) Effect of PV treatment on SARS‐CoV‐2 RNA levels from cell supernatants using qRT‐PCR. (i) Cytotoxicity of PV in HBEpCs from the apical medium measured via LDH release assay. *p*‐values were calculated via Brown–Forsythe test (e), Kruskal–Wallis test (f) or ordinary one‐way ANOVA ((h) and (i)) and are indicated for each significant group compared to the untreated virus control. Results are expressed as the mean ± SD.

Next, we investigated the PV's mediated antiviral effect against SARS‐CoV‐2. We first explored if PV retains its activity also against different SARS‐CoV‐2 variants (Figure 3a). Consistently, all three compounds exhibited similar dose–response curves across all tested variants, with IC_50_ values ranging from 1.9 to 3.9 µM for PV, 3.1 to 4.8 µM for EV, and only limited inhibition by DV, aligning with previous findings (Figure 3b,c).

Given that PV targets PDE activity, we evaluated other PDE inhibitors for anti‐SARS‐CoV‐2 activity. Testing PDE4‐ and 10A‐specific inhibitors, as well as a nonselective PDE inhibitor, revealed no antiviral activity (Figure S1), suggesting that the antiviral effects of PV and its derivatives are independent of their PDE inhibitory activity.

To decipher the stage at which these drugs act in the viral life cycle, we infected Calu‐3 cells with the SARS‐CoV‐2 Delta variant and treated them with PV, EV, DV, or nafamostat (NAF), a known inhibitor of TMPRSS2‐mediated viral entry [27] (Figure 3d). The treatment was administered either during or post‐virus entry, and the infection rate was evaluated after a single replication cycle (approximately 8 h). Treatment during virus entry with PV, EV, or DV resulted in only marginal inhibition, mostly nonsignificant, whereas NAF significantly decreased viral RNA levels (Figure 3e). Conversely, post‐entry treatment with PV and EV notably reduced SARS‐CoV‐2 levels, while DV did not show significant inhibition consistent with its lower potency (Figure 3f). As anticipated, NAF exhibited no inhibitory effect when added post‐entry.

Subsequently, we assessed the antiviral efficacy and cellular toxicity of PV in HBEpCs cultured under ALI conditions (Figure 3g). A significant reduction in the release of viral RNA levels was observed following treatment with both 5 and 0.5 µM PV (Figure 3h). No compound‐induced toxicity, as measured by LDH release, was detected in either uninfected or infected cells; on the contrary, 5 µM PV treatment in infected cells resulted in a slight but significant decrease in cytotoxicity (Figure 3i).

### 3.4 PV Inhibits IFN Signaling Independently of Virus Infection

**Figure 4:**
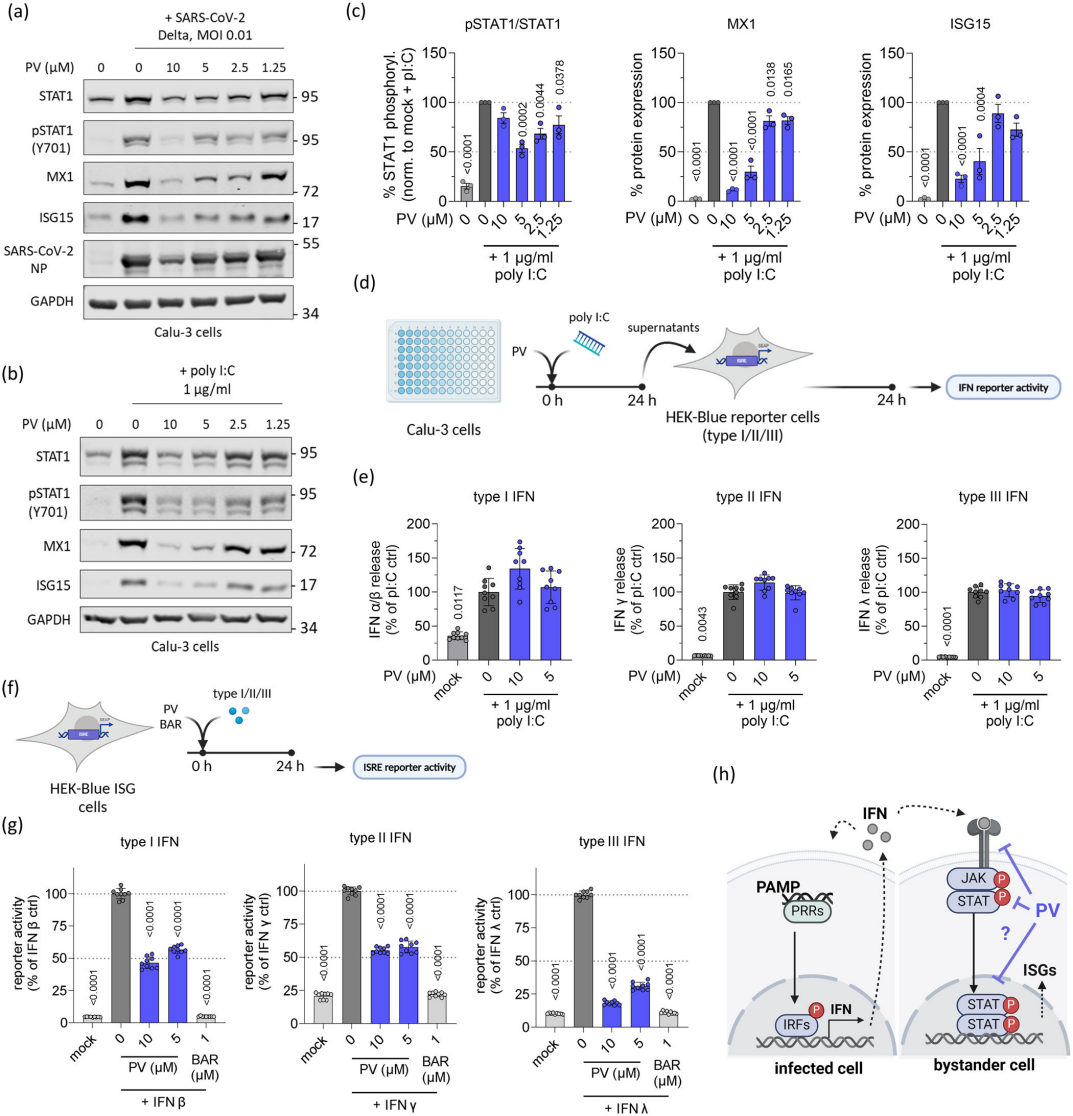
PV inhibits IFN signaling independently of virus infection. (a) and (b) Representative immunoblot images of Calu‐3 cells treated with different concentrations of PV and either infected with SARS‐CoV‐2 (Delta variant) (a) or stimulated with 1 µg/mL poly I:C (b). (c) Densitometric quantification and statistic evaluation of the bands in (b). (d) Experimental scheme of interferon release measurements. Calu‐3 cells were treated with PV and stimulated with poly I:C for 24 h, and the cell supernatant subsequently incubated with HEK‐Blue reporter cells for IFN type I, II, or III, respectively. After 24 h, interferon release was determined via IFN reporter activity measurement (e). (f) Experimental scheme of interferon stimulated response element (ISRE) activation assay. HEK‐Blue ISG reporter cells were treated with PV or baricitinib (BAR) and stimulated with IFN type I, II, or III, respectively. Inhibition of ISRE activation was determined after 24 h (g). (h) Scheme of potential targets of PV's effect on innate immune activation. *p*‐values were calculated via ordinary one‐way ANOVA and are indicated for each significant group compared to the untreated virus‐ or poly I:C‐control, respectively. Results are expressed as the mean ± SD.

Due to PV's previously reported immunomodulatory effects [9, 10, 11], we examined its impact on innate immune response in Calu‐3 cells after both SARS‐CoV‐2 infection and stimulation with poly I:C. We observed a dose‐dependent reduction in STAT1 phosphorylation and the expression of MX1 and ISG15 in both virus‐infected (Figure 4a) and poly I:C‐stimulated cells (Figure 4b,c). Notably, a similar inhibitory effect was observed with EV treatment, while DV treatment produced a significantly weaker response (Figure S2), mirroring the patterns seen in the viral infection experiments.

To further dissect the specific aspect of IFN signaling affected by PV, we examined its effect on ISRE and NFκB promoter activity in A549 cells, both wild‐type and knockout for the RIG‐I and MDA5 receptors, which are key dsRNA sensors and initiators of IFN signaling (Figure S3a). PV significantly reduced ISRE and NFκB activation regardless of receptor knockout (Figure S3b,c), suggesting that PV does not target these initial sensors.

Importantly, when measuring type I, II, and III IFN release in the supernatants of PV‐treated cells using reporter assays (Figure 4d), we observed no significant changes in IFN levels for any of the three types (Figure 4e). Furthermore, when reporter cells stimulated with type I‐III IFNs were treated with PV (Figure 4f), there was a marked inhibition of ISRE activation, with reductions of approximately 50% for type I and II IFNs and up to 75% for type III IFNs (Figure 4g). These findings collectively suggest that PV interferes with IFN signaling downstream of IFN release, for example, through direct prevention of IFN‐receptor interaction or JAK/STAT signaling block (Figure 4h).

### 3.5 PV Inhibits STAT1/2 Nuclear Translocation

**Figure 5:**
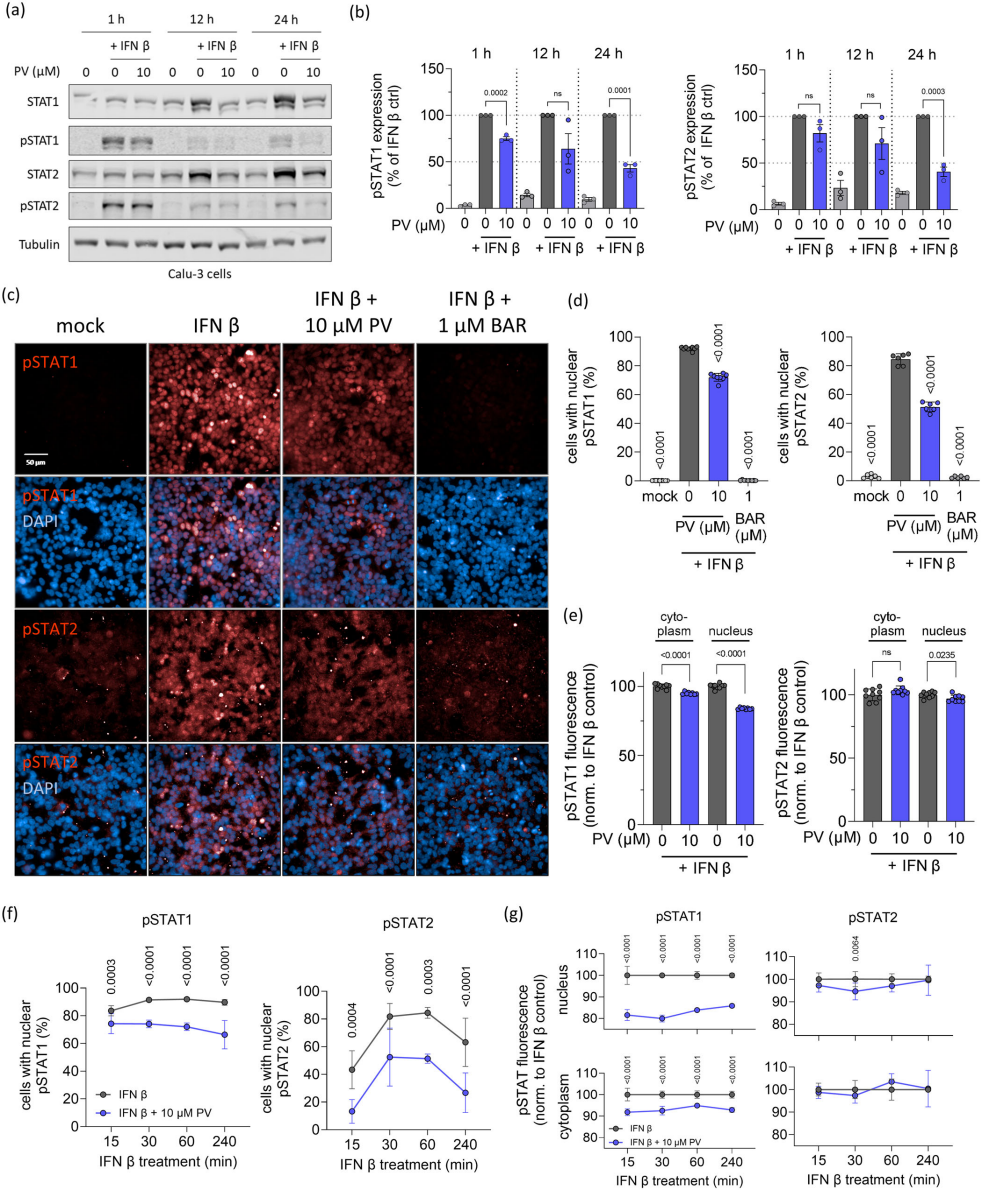
Papaverine inhibits phosphorylation and nuclear translocation of pSTAT1 and pSTAT2 in Calu‐3 and HEK‐Blue ISG cells. (a) Representative immunoblot images of Calu‐3 cells treated with PV and stimulated with IFN β for 1, 12, or 24 h. (b) Densitometric quantification and statistic evaluation of the pSTAT1 and pSTAT2 bands in (a). (c) Representative immunofluorescence pictures of HEK‐Blue ISG reporter cells treated with PV or BAR and stimulated with IFN β for 1 h. The inhibition of pSTAT1/2 activation and translocation was determined through the percentage of cells with nuclear pSTAT1/2 (d) and fluorescence intensity of pSTAT1/2 in cells with nuclear signal (e), each compared to the respective IFN β control. (f) and (g) Cells with nuclear pSTAT1/2 and fluorescence intensity of pSTAT1/2 in cells with nuclear signal over time. *p*‐values were calculated via unpaired *t*‐test ((b) and (e)), Brown–Forsythe test (d) or two‐way ANOVA ((f) and (g)) and are indicated for each significant group compared to the respective untreated IFN β control. Results are expressed as the mean ± SD.

Given that each type of IFN signals through distinct receptors, it is unlikely that PV targets these receptors directly. Therefore, we focused on the downstream signaling events involving STAT1 and STAT2, which are shared across all IFN types. Immunoblot analyses of STAT1 and STAT2 phosphorylation in cells treated with IFN β and PV revealed a significant reduction in phosphorylation levels after 24 h (Figure 5a,b). However, at earlier time points (1 and 12 h), PV had only a marginal effect on pSTAT1 and pSTAT2 levels, suggesting that the reduction in phosphorylation might be a secondary effect of PV treatment.

To investigate this further, we examined the nuclear translocation of phosphorylated STAT1/2 1 h post‐IFN β stimulation (Figure 5c). PV treatment led to a significant decrease in the percentage of cells with nuclear pSTAT1 or pSTAT2 signals, reducing these populations by 22% and 29%, respectively (Figure 5d). Among the cells with nuclear pSTAT1/2 signals, PV treatment caused a more pronounced reduction in fluorescence intensity within the nucleus compared to the cytoplasm for both pSTAT1 and pSTAT2 (Figure 5e). Notably, the decrease in the percentage of cells with nuclear pSTAT1/2 was evident at different time points, appearing as early as 15 min after treatment and persisting up to 4 h for both pSTAT1 and pSTAT2 (Figure 5f). Similarly, pSTAT1 fluorescence was reduced in both the nucleus and cytoplasm throughout all time points from 15 min to 4 h, while a slight but significant reduction in pSTAT2 fluorescence was observed in the nucleus after 30 min of PV treatment (Figure 4g).

### 3.6 PV Exhibits Synergistic Potential in Combination With Other Anti‐SARS‐CoV‐2 Drugs

**Figure 6:**
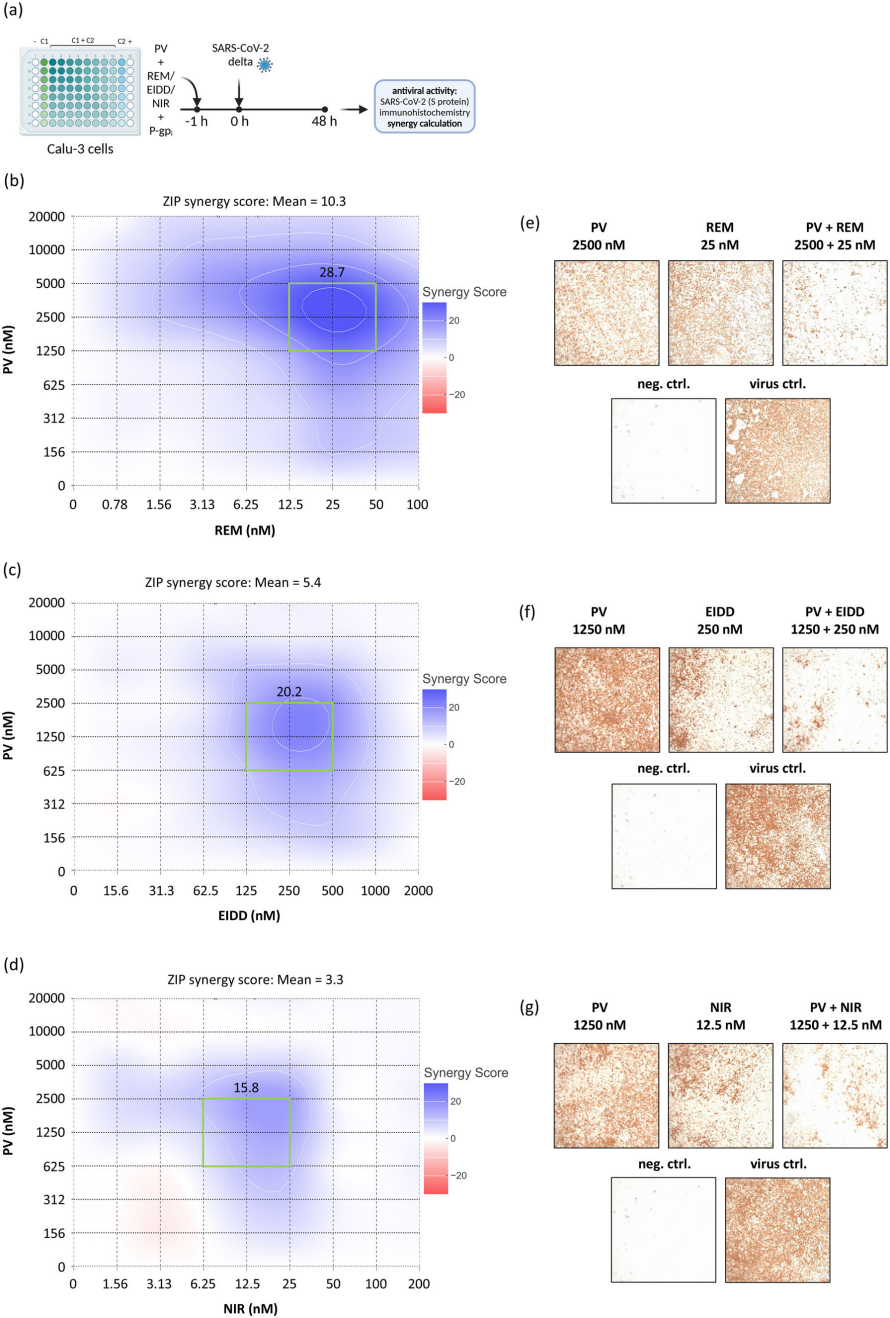
Papaverine indicates potential for combination therapy with other anti‐SARS‐CoV‐2 drugs. (a) Experimental setup of the combination experiments. Calu‐3 cells were treated with PV and remdesivir (REM), EIDD‐1931 (EIDD) or nirmatrelvir (NIR), respectively, in different concentration combinations and subsequently infected with SARS‐CoV‐2 (Delta variant). (b)–(d) Synergy distribution matrices and mean ZIP synergy score across all concentrations of combination treatments with PV and REM, EIDD, or NIR, respectively. A ZIP score of > 10 indicates synergistic action of the tested compounds. The green rectangle denotes the highest synergistic area with its respective synergy score. The corresponding percentages of inhibition and the synergy scores for each combination can be found in Figures S4 and S5. (e) and (g) Representative pictures of the effect of the drug combinations in the highest synergistic area's center concentrations. Results are expressed as the mean of three independent experiments.

Combining antiviral drugs is crucial for enhancing treatment efficacy and reducing the likelihood of drug resistance [28, 29]. To explore the potential of PV in combination therapy for SARS‐CoV‐2, we conducted drug combination assays pairing PV with REM, EIDD (Molnupiravir), and NIR (Paxlovid) (Figure 6a). All combinations demonstrated a trend toward synergistic effects, as indicated by ZIP synergy scores above 10 – specifically, 10.3 for REM, 5.4 for EIDD, and 3.3 for NIR (Figures 6b–d and S4). Notably, each drug pairing showed areas of pronounced synergy (REM: 28.7, EIDD: 20.2, NIR: 15.8) (Figures 6b–d and S5), which also is reflected by immunohistological staining (Figure 6e–g). These findings suggest that PV enhances the efficacy of existing anti‐SARS‐CoV‐2 therapeutics through synergistic interactions.

## 4 Discussion

In this study, we demonstrated a dual effect of PV, the inhibition of both viral infection and IFN signaling. First, we showed the dose‐response antiviral activity of PV and its derivatives EV and DV against different SARS‐CoV‐2 variants as well as IAV H1N1 and H5N1. Owing to the strongly reduced antiviral activity of DV against both SARS‐CoV‐2 and IAV compared to PV or EV (Figure 1b), as well as EV's cytotoxicity at high doses (Figure 1d), we identified PV as the most promising candidate of the three tested compounds and focused further studies on it. These data, together with the reported antiviral effects of PV on other viruses as different paramyxoviruses or respiratory syncytial virus [12], highlight PV's potential as a broad‐spectrum antiviral substance. Additionally, PV's sustained potency against both viruses in a SARS‐CoV‐2 and IAV co‐infection model could be valuable in treating this clinically relevant co‐infection, which has been associated with an increased risk of death or the increased need for mechanical ventilation [26].

The antiviral effects of PV were observed in both colon and lung cancer cell lines as well as the primary respiratory epithelium cell model, suggesting that its mechanism of action is not specific to a particular cell type or cancerous tissue. Additionally, all tested virus variants showed similar dose‐response curves and IC_50_‐values after PV treatment (Figure 3), indicating a mode of action independent of the most variable structure of the virus – its spike protein [30]. This was further validated in time‐of‐addition experiments, which demonstrated that PV's antiviral effect occurs in a post‐entry phase of the replication cycle (Figure 3). As a result, PV can be used as a treatment for an existing SARS‐CoV‐2 infection, rather than for prophylactic administration.

Secondly, we could show that treatment with PV resulted in reduction of IFN signaling, which is often deregulated in severe cases of COVID‐19 [2]. PV has two known targets – PDE and mitochondrial respiratory complex I [7]. PV‐mediated inhibition of PDE is known to elevate intracellular levels of both cAMP and cGMP, key regulators of signaling pathways [7]. Elevated cAMP levels have been shown to suppress STAT1 DNA binding in mononuclear and T cells [31]. Additionally, cAMP activates PKA, which has been reported to inhibit type I IFN signaling [32] and has also been required for PV‐induced suppression of the NFκB pathway [9]. In addition to PDE inhibition, Benej et al. reported a decrease in mitochondrial respiration during PV treatment. They demonstrated that this effect is mediated by PV's targeting of respiratory complex I and that the inhibition occurs independently of its PDE activity. Interestingly, the suppression of mitochondrial respiration by rotenone, an inhibitor of respiratory complex I, as well as the knockdown of NDUFV1, an essential subunit of complex I, has been shown to suppress JAK/STAT signaling in IFNγ‐treated cells [33]. Therefore, the observed inhibition of STAT1 and STAT2 signaling is more likely a consequence of PV‐driven intracellular signaling via cAMP elevation and perturbation of mitochondrial respiration than direct interaction with STAT proteins. However, the involvement of PDE‐ and mitochondrial respiration‐independent mechanisms cannot be ruled out.

Collectively, our findings suggest that PV modulates inflammatory responses via dual mechanisms targeting both NFκB and JAK/STAT pathways, potentially via modulation of cAMP levels and/or mitochondrial respiration. Given that both these pathways are potential targets for COVID‐19 interventions [34, 35, 36], the broad range of PV's effects may offer valuable benefits in mitigating the disease as a whole. Particularly, targeting JAK/STAT signaling has already been successfully employed in COVID‐19 therapy. BAR, a JAK inhibitor, has received FDA approval for use, but lacks antiviral activity, requiring combination therapy with the direct‐acting antiviral REM [37, 38]. Without this combination, there is a risk of enhancing viral infection by blocking the induction of a cellular antiviral state [39]. In contrast, PV may offer the added benefit of both acting as an antiviral agent and modulating the innate immune system. Further, its favorable performance in the drug synergy experiments (Figure 6) supports the promising option for combination therapy, potentially with lower individual doses, as combining antiviral drugs was shown to be crucial for enhancing treatment efficacy and reducing the likelihood of drug resistance [28, 29].

PV has been reported to have an excellent safety profile in patients, with injection site fibrosis after intravenous or intramuscular application being the most common long‐term side effect [40]. Although off‐target effect such as mitochondrial complex I inhibition was observed in vitro [7], this inhibition was reversible. Furthermore, due to its extensive metabolization and short plasma half‐life, no accumulation of the drug has been observed [41]. Therefore, long‐term or irreversible immunosuppressive effects of PV are rather unlikely. Nevertheless, preclinical studies are still necessary to rule out any potential long‐term immunomodulatory consequences, particularly in specific patient populations with underlying immune dysfunction.

In conclusion, this study revealed PV's dual action in inhibiting SARS‐CoV‐2 and IAV infection and downregulating IFN‐induced JAK/STAT signaling. Since similar immunomodulatory and antiviral effects were also observed with PV derivative, EV, and to a lower extent, also with DV, it suggests that these effects may be characteristic of the entire class of structurally similar drugs rather than PV alone. Further research into other existing or newly synthesized compounds with similar structures is necessary to fully explore and harness the potential of these dual effects. Additionally, PV's activity against various respiratory viruses and favorable performance in drug combination experiments highlight its potential in combination therapies for complex co‐infections in clinical settings.

## Author Contributions

**Philipp Reus:** methodology, validation, formal analysis, investigation, visualization, writing – original draft. **Emma Torbica:** methodology, validation, investigation, formal analysis, writing – original draft. **Tamara Rothenburger:** methodology, validation, investigation, formal analysis, writing – review and editing. **Marco Bechtel:** methodology, validation, investigation. **Joshua Kandler:** methodology, validation, formal analysis. **Sandra Ciesek:** supervision, funding acquisition. **Philip Gribbon:** conceptualization, supervision. **Aimo Kannt:** conceptualization, supervision. **Jindrich Cinatl:** conceptualization, supervision, project administration, funding acquisition. **Denisa Bojkova:** conceptualization, methodology, validation, formal analysis, supervision, project administration, funding acquisition, writing – review and editing.

## Conflicts of Interest

The authors declare no conflicts of interest.

## Supporting information

Supporting information.

## Data Availability

Data are available upon request.
