# Supplementary material for: Papaverine Targets STAT Signaling: A Dual‐Action Therapy Option Against SARS‐CoV‐2
Source: J Med Virol. 2025 Apr 2;97(4):e70319. doi: 10.1002/jmv.70319 (PMC11963225; doi:10.1002/jmv.70319)
Supplement: Supplementary file 1 — Supporting information. [file JMV-97-e70319-s001.pptx]

## Slide 1
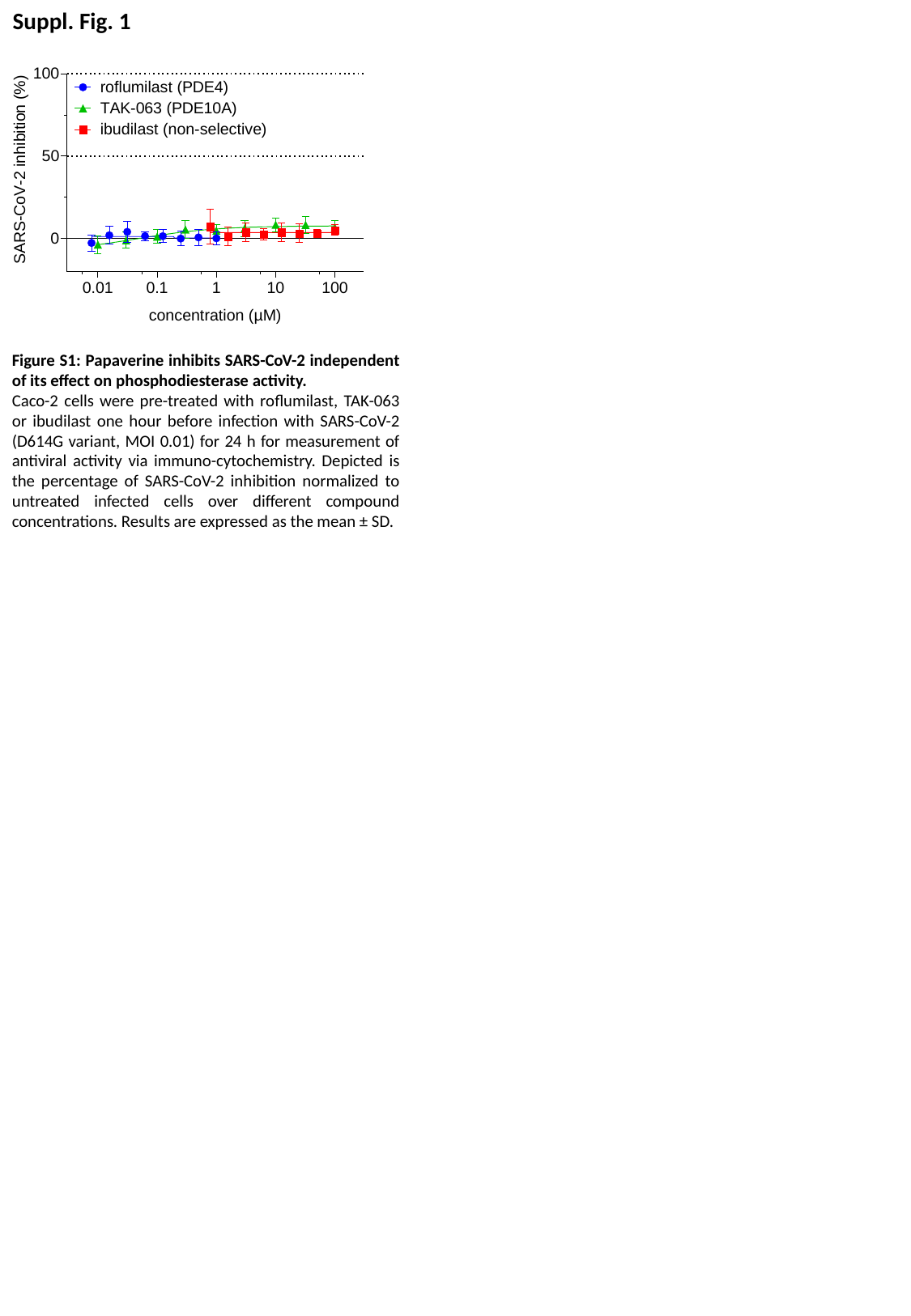

Suppl. Fig. 1
Figure S1: Papaverine inhibits SARS-CoV-2 independent of its effect on phosphodiesterase activity.
Caco-2 cells were pre-treated with roflumilast, TAK-063 or ibudilast one hour before infection with SARS-CoV-2 (D614G variant, MOI 0.01) for 24 h for measurement of antiviral activity via immuno-cytochemistry. Depicted is the percentage of SARS-CoV-2 inhibition normalized to untreated infected cells over different compound concentrations. Results are expressed as the mean ± SD.

## Slide 2
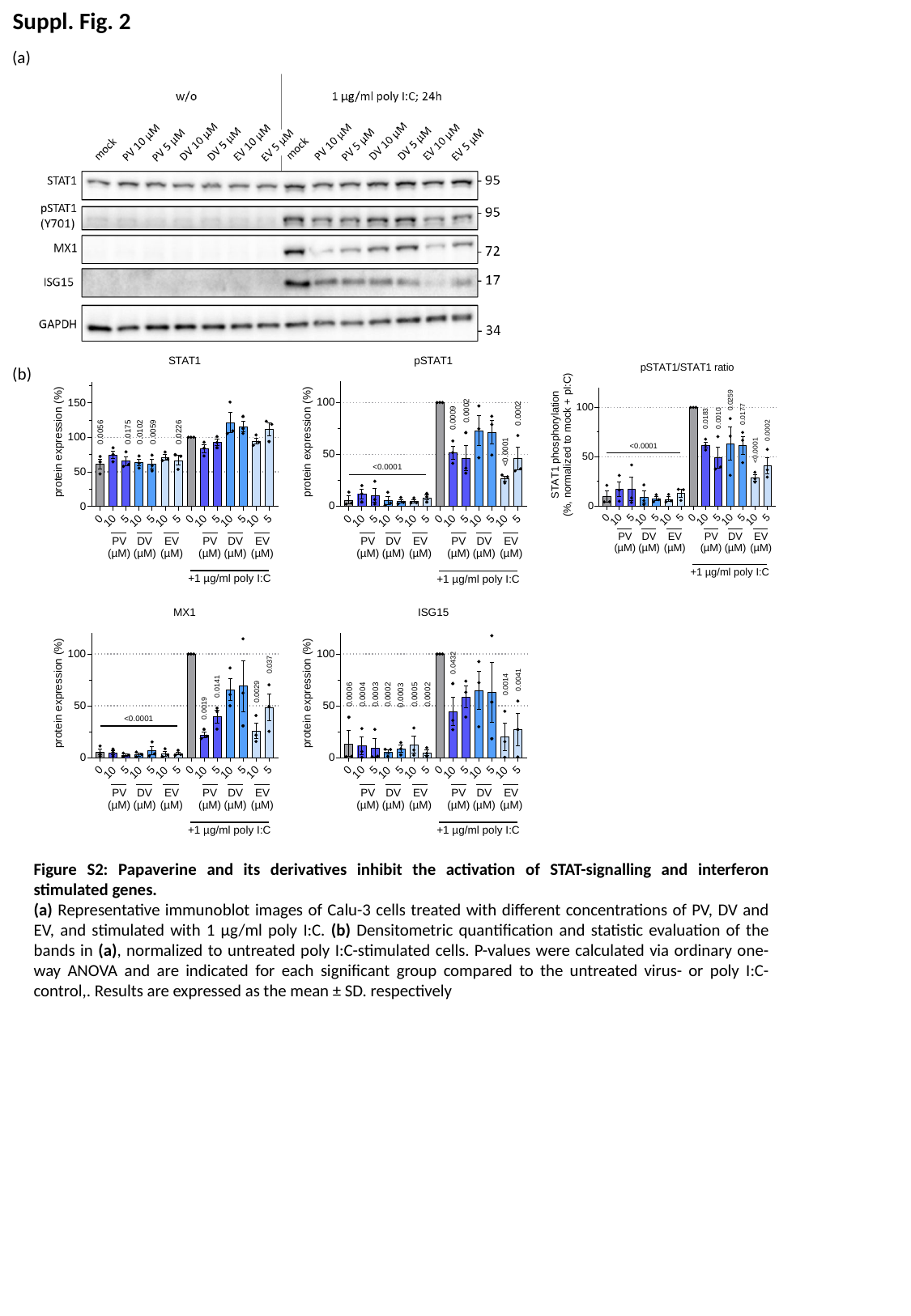

Suppl. Fig. 2
(a)
(b)
Figure S2: Papaverine and its derivatives inhibit the activation of STAT-signalling and interferon stimulated genes.
(a) Representative immunoblot images of Calu-3 cells treated with different concentrations of PV, DV and EV, and stimulated with 1 µg/ml poly I:C. (b) Densitometric quantification and statistic evaluation of the bands in (a), normalized to untreated poly I:C-stimulated cells. P-values were calculated via ordinary one-way ANOVA and are indicated for each significant group compared to the untreated virus- or poly I:C-control,. Results are expressed as the mean ± SD. respectively

## Slide 3
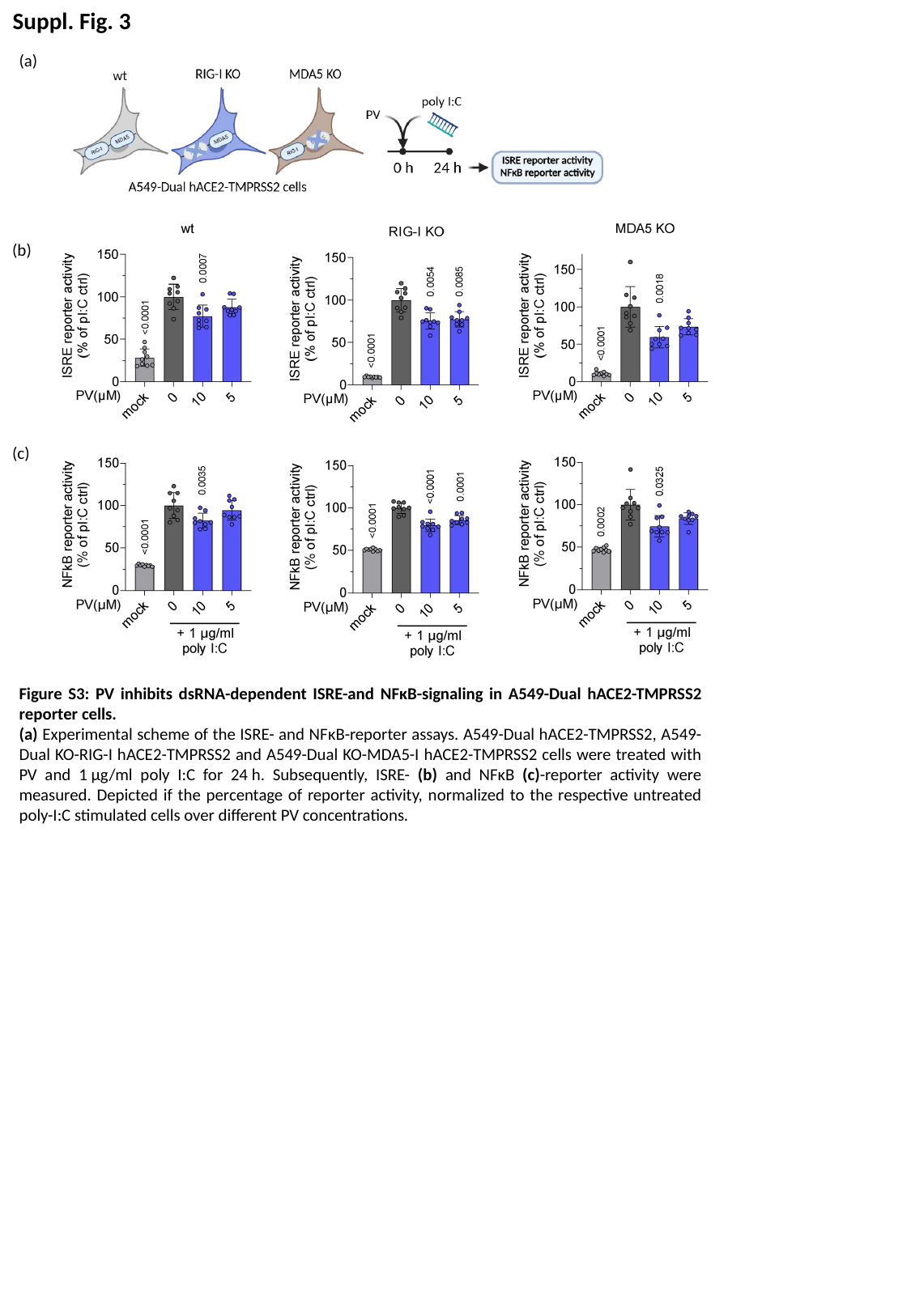

Suppl. Fig. 3
(a)
(b)
(c)
Figure S3: PV inhibits dsRNA-dependent ISRE-and NFκB-signaling in A549-Dual hACE2-TMPRSS2 reporter cells.
(a) Experimental scheme of the ISRE- and NFκB-reporter assays. A549-Dual hACE2-TMPRSS2, A549-Dual KO-RIG-I hACE2-TMPRSS2 and A549-Dual KO-MDA5-I hACE2-TMPRSS2 cells were treated with PV and 1 µg/ml poly I:C for 24 h. Subsequently, ISRE- (b) and NFκB (c)-reporter activity were measured. Depicted if the percentage of reporter activity, normalized to the respective untreated poly-I:C stimulated cells over different PV concentrations.

## Slide 4
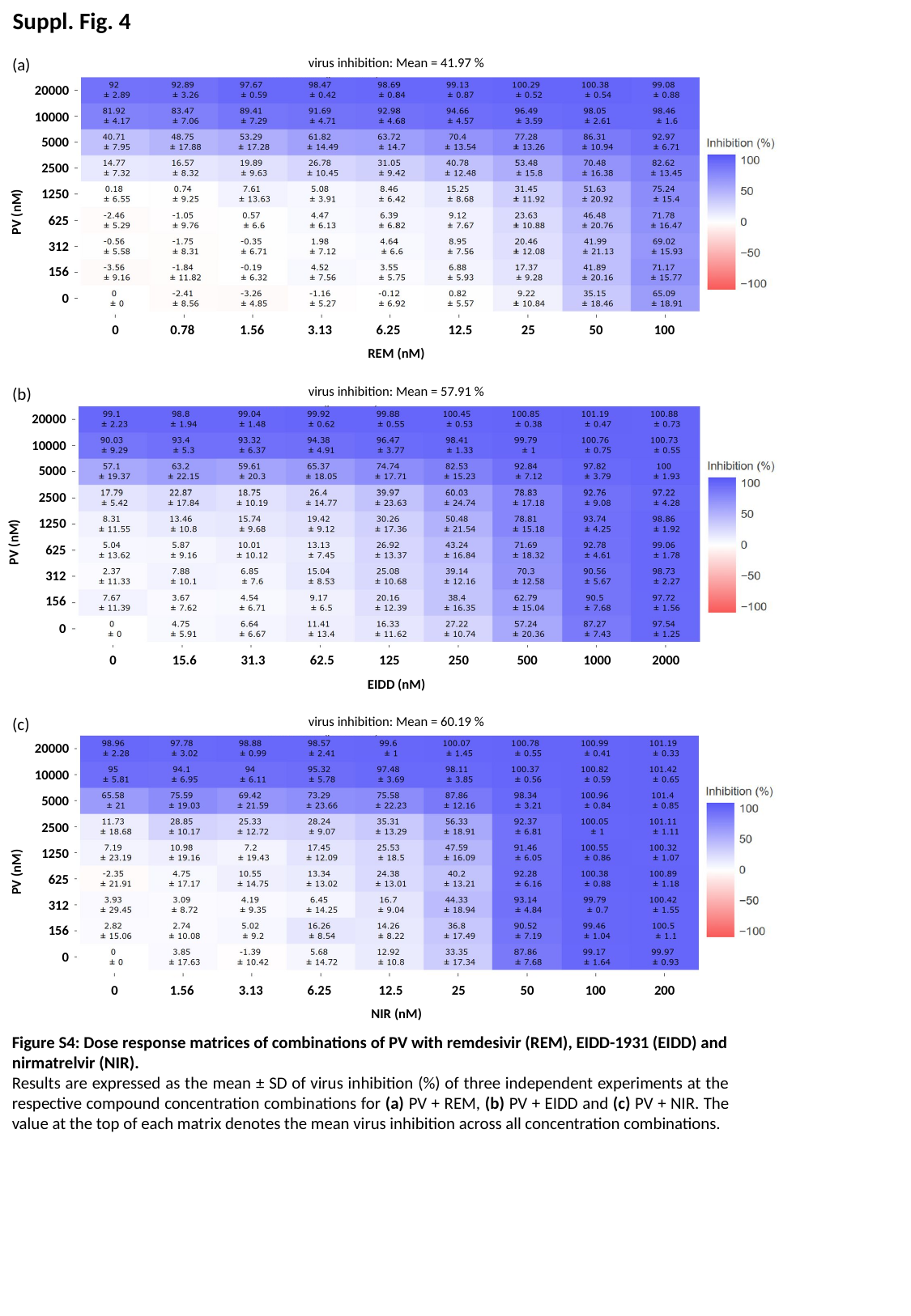

Suppl. Fig. 4
(a)
virus inhibition: Mean = 41.97 %
20000
10000
5000
2500
1250
625
312
156
0
PV (nM)
0
0.78
1.56
3.13
6.25
12.5
25
50
100
REM (nM)
(b)
virus inhibition: Mean = 57.91 %
20000
10000
5000
2500
1250
625
312
156
0
PV (nM)
0
15.6
31.3
62.5
125
250
500
1000
2000
EIDD (nM)
(c)
virus inhibition: Mean = 60.19 %
20000
10000
5000
2500
1250
625
312
156
0
PV (nM)
0
1.56
3.13
6.25
12.5
25
50
100
200
NIR (nM)
Figure S4: Dose response matrices of combinations of PV with remdesivir (REM), EIDD-1931 (EIDD) and nirmatrelvir (NIR).
Results are expressed as the mean ± SD of virus inhibition (%) of three independent experiments at the respective compound concentration combinations for (a) PV + REM, (b) PV + EIDD and (c) PV + NIR. The value at the top of each matrix denotes the mean virus inhibition across all concentration combinations.

## Slide 5
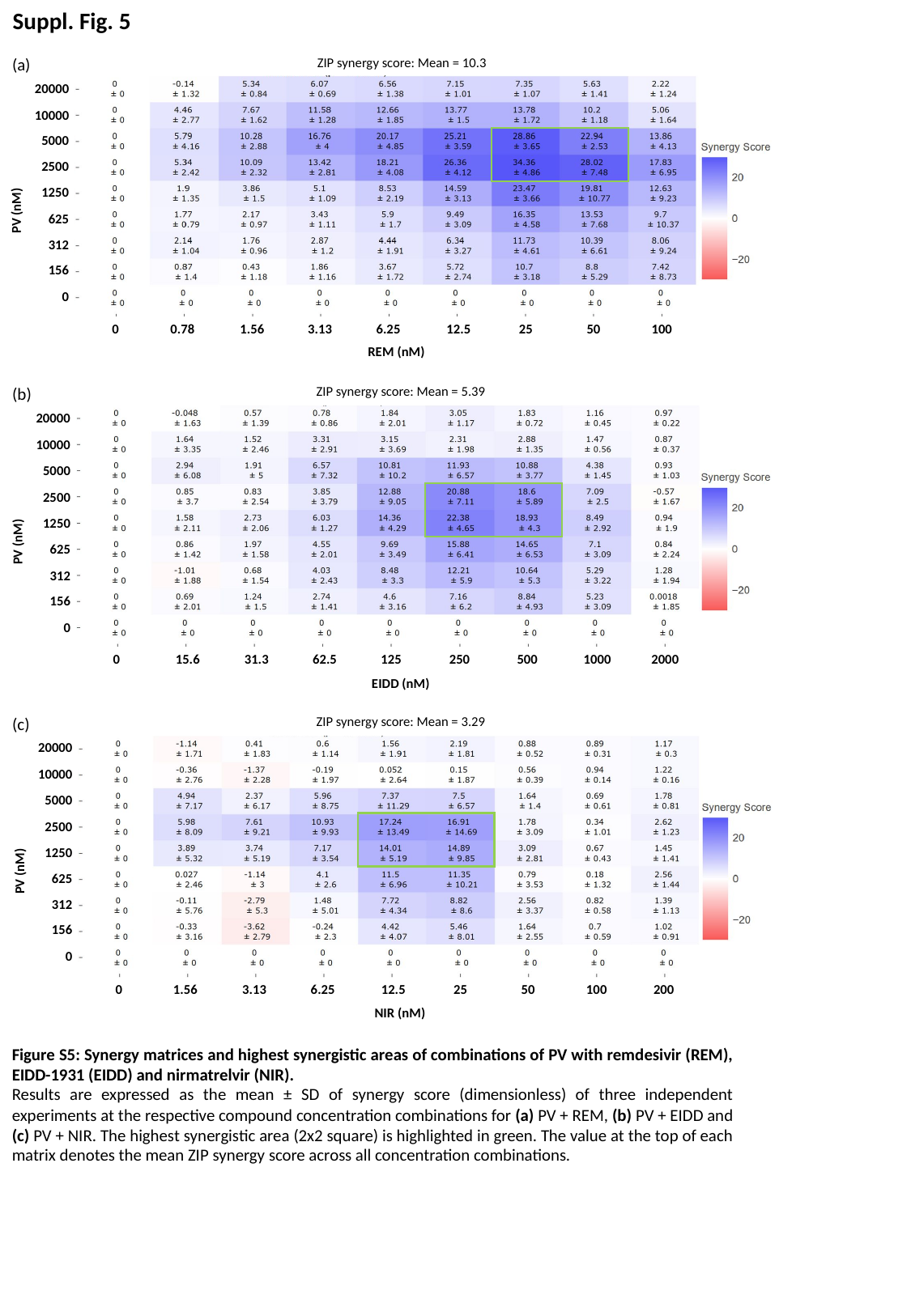

Suppl. Fig. 5
(a)
ZIP synergy score: Mean = 10.3
20000
10000
5000
2500
1250
625
312
156
0
PV (nM)
0
0.78
1.56
3.13
6.25
12.5
25
50
100
REM (nM)
(b)
ZIP synergy score: Mean = 5.39
20000
10000
5000
2500
1250
625
312
156
0
PV (nM)
0
15.6
31.3
62.5
125
250
500
1000
2000
EIDD (nM)
(c)
ZIP synergy score: Mean = 3.29
20000
10000
5000
2500
1250
625
312
156
0
PV (nM)
0
1.56
3.13
6.25
12.5
25
50
100
200
NIR (nM)
Figure S5: Synergy matrices and highest synergistic areas of combinations of PV with remdesivir (REM), EIDD-1931 (EIDD) and nirmatrelvir (NIR).
Results are expressed as the mean ± SD of synergy score (dimensionless) of three independent experiments at the respective compound concentration combinations for (a) PV + REM, (b) PV + EIDD and (c) PV + NIR. The highest synergistic area (2x2 square) is highlighted in green. The value at the top of each matrix denotes the mean ZIP synergy score across all concentration combinations.
